# GPR55 is expressed in glutamate neurons and functionally modulates nicotine taking and seeking in rats and mice

**DOI:** 10.21203/rs.3.rs-3222344/v1

**Published:** 2023-10-03

**Authors:** Zheng-Xiong Xi, Yi He, Hui Shen, Guo-hua Bi, Hai-Ying Zhang, Omar Soler-Cedeno, Hannah Alton, Yihong Yang

**Affiliations:** National Institute on Drug Abuse; National Institute on Drug Abuse; National Institute on Drug Abuse; National Institute on Drug Abuse; National Institute on Drug Abuse; National Institute on Drug Abuse; National Institute on Drug Abuse; National Institute on Drug Abuse

**Keywords:** GPR55, Δ9-tetrahydrocannabinol, cocaine, nicotine, self-administration, reward, dopamine neurons, glutamate neurons

## Abstract

Cannabis legalization continues to progress in the USA for medical and recreational purposes. G protein-coupled receptor 55 (GPR55) is a putative “CB3” receptor. However, its functional role in cannabinoid action and drug abuse is not explored. Here we report that GPR55 is mainly expressed in cortical and subcortical glutamate neurons and its activation attenuates nicotine taking and seeking in rats and mice. RNAscope *in situ* hybridization detected GPR55 mRNA in cortical vesicular glutamate transporter 1 (VgluT1)-positive and subcortical VgluT2-positive glutamate neurons in wildtype, but not GPR55-knockout, mice. GPR55 mRNA was not detected in midbrain dopamine (DA) neurons in either genotype. Immunohistochemistry assays detected GPR55-like staining, but the signal is not GPR55-specific as the immunostaining was still detectable in GPR55-knockout mice. We then used a fluorescent CB1-GPR55 ligand (T1117) and detected GPR55 binding in cortical and subcortical glutamate neurons, but not in midbrain DA neurons, in CB1-knockout mice. Systemic administration of O-1602, a GPR55 agonist, dose-dependently increased extracellular glutamate, not DA, in the nucleus accumbens. Pretreatment with O-1602 failed to alter Δ^9^-tetrahydrocannabinol (D^9^-THC)-induced triad effects or intravenous cocaine self-administration, but it dose-dependently inhibited nicotine self-administration under fixed-ratio and progressive-ratio reinforcement schedules in rats and wildtype mice, not in GPR55-knockout mice. O-1602 itself is not rewarding or aversive as assessed by optical intracranial self-stimulation (oICSS) in DAT-Cre mice. These findings suggest that GPR55 is functionally involved in nicotine reward process possibly by a glutamate-dependent mechanism, and therefore, GPR55 deserves further research as a new therapeutic target for treating nicotine use disorder.

The endocannabinoid system is implicated in numerous physiological functions, such as emotion, locomotion, cognition, learning and memory, as well as cannabis action and drugs of abuse^1–5^. Extensive research in the past several decades indicates that cannabinoid CB1 and CB2 receptors are the major receptors in the brain involved in the CNS effects of cannabinoids^5, 6^. However, cannabinoids also bind to other non-CB1 and non-CB2 receptors such as G protein-coupled receptor 55 (GPR55)^5, 7^, while little is known about the functional role of GPR55 in cannabinoid action and other drug abuse.

GPR55 was first isolated in 1999 as an orphan GPCR with high expression levels in the human striatum^8^. Initially, it was identified as a potential purinergic or chemokine-like receptor based on amino acid homology^8^. However, further studies indicate that GPR55 could be a cannabinoid “CB3” receptor because multiple cannabinoids, including the endocannabinoids [anandamide (AEA) and 2-arachidonoylglycerol (2-AG)], phytocannabinoid [Δ^9^-tetrahydrocannabinol (D^9^-THC)], and synthetic cannabinoids (HU-210, CP55,940), can bind and activate GPR55^7, 9^. Lysophosphatidylinositol (LPI) has been identi ed as an endogenous ligand of GPR55^10, 11^. Structurally, GPR55 is distinct from both CB1 and CB2 receptors because it lacks the classical endocannabinoid binding pocket^12^ and has minimal receptor homology with CB1 (13.5%) or CB2 receptors (14.4%)^13^.

Quantitative RT-PCR assays revealed GPR55 gene expression in the mouse brain, including the frontal cortex, striatum, hippocampus, and cerebellum^9, 14^. In situ hybridization (ISH) and immunohistochemistry (IHC) assays also show GPR55 mRNA and immunostaining signals in neurons in the striatum and hippocampus^15–18^. However, the detected GPR55 signals are very weak and the phenotypes of neurons that express GPR55 in the striatum and hippocampus are largely unknown. It is also unknown whether functional GPR55 is expressed on cortical glutamate neurons and midbrain DA neurons.

Functionally, GPR55 has been shown to modulate anxiety-related behavior^19, 20^, pain perception^21^, locomotion^14, 22^, learning and memory^23^, and the conditioned place preference (CPP) response to morphine or nicotine^24–26^. In addition, we recently reported that genetic deletion of GPR55 enhanced behavioral responses to the cannabinoids D^9^-THC and WIN55,212–2 in analgesia, hypothermia, and catalepsy tests^22^, suggesting that GPR55 activation may functionally inhibit the triad effects of cannabinoids mediated by activation of CB1 and CB2 receptors. These findings suggest that GPR55 agonists may have therapeutic effects against cannabinoid or other substance use disorders. However, direct evidence is lacking.

In this study, we first used multiple neuroimaging approaches, including advanced RNAscope ISH, IHC, and fluorescent ligand binding assays, to examine the GPR55 gene/receptor expression in brain DA and glutamate neurons. We then examined whether systemic administration of O-1602, a potent GPR55 agonist (with EC_50_ values of 13, > 30,000, and > 30,000 nM for GPR55, CB1 and CB2 receptors, respectively)^9^, alters DA or glutamate release in the nucleus accumbens (NAc) in rats using in vivo microdialysis. Next, we examined the effects of O-1602 pretreatment on D^9^-THC-induced triad effects (analgesia, hypothermia and catalepsy) and on intravenous cocaine or nicotine self-administration in rats and mice. We also examined the effects of O-1602 on oral sucrose self-administration and open-field locomotion to determine whether O-1602 also alters non-drug reward or locomotor impairment. Lastly, we used optical brain-stimulation reward to determine whether O-1602 itself produces reward-enhancing (rewarding) or reward-attenuation (aversive) effects. GPR55-knockout (GPR55-KO) mice were used as negative controls to determine GPR55 signal specificity and O-1602 pharmacological specifciity. We found that GPR55 is mainly expressed in glutamate neurons in the brain and functionally modulates nicotine self-administration.

## Methods and Materials

Male adult Long-Evans (LE) and alcohol-preferring rats (P-rats, obtained from Indiana University Center) were used in the present study. Male and female GPR55-KO mice were purchased from Lexicon Pharmaceuticals (Cat. #: LEXKO-0261, B6;129S-Gpr55^tm1Lex^/Mmnc, The Woodlands, Texas, USA).Heterozygous CB_1_+/− breeding pairs were provided by Dr. Andreas Zimmer during his tenure at the National Institute of Mental Health (Bethesda, MD). DAT-cre mice (Slc6a3tm1(cre)Xz/J) were obtained from the Jackson Laboratory (Stock No: 020080). All of the transgenic mice and their wild-type littermates were bred in the animal facility of the National Institute on Drug Abuse (NIDA) Intramural Research Program (IRP). The animal care and the experimental procedure were conducted in accordance with the Guide for the Care and Use of Laboratory Animals and were approved by the Animal Care and Use Committee of NIDABoth male rats were used in the present study. The full descriptions about the experimental animals, the experimental methods, and the data analysis are provided in *Supplmentary Information*.

### Chemicals

Cocaine, nicotine and Δ^9^-THC was provided by the NIDA pharmacy (Baltimore, MD). O-1602 was supplied by Cayman Chemical (Cat#: 10006803). The selective GPR55 antagonist CID 16020046 (CID, Cat#: 4959) and Tocrifluor T1117 were purchased from Tocris Bioscience. For the more in detail, please see *Supplmentary Information*.

### Experiment 1

#### RNAscope in situ hybridization

We first performed RNAscope *in situ* hybridization (ISH) to examine the distribution of GPR55 mRNA in the cortex and subcortical brain regions including hippocampus, thalamus, VTA and red nucleus (RN) and striatum. The complete RNAscope procedures are described in *Supplementary Information*.

### Experiment 2: Immunohistochemistry (IHC)

RNAscope ISH assays detected GPR55 mRNA in cortical and subcortical glutamate neurons. To further confirm this finding, we used double label IHC to examine GPR55-immunostaining. The complete IHC procedures are described in *Supplementary Information*.

### Experiment 3: Flfluorescent GPR55-ligand binding assays

Given that the GPR55 antibody displayed poor receptor specificity(see the results section below), we next used a novel fluorescent ligand (Tocrifluor T1117, i.e., T1117) to examine T1117 binding on GPR55^27, 28^.CB1-KO mice were used in this assay because T1117 is a fluorescent analog of AM251 (a selective CB1 receptor antagonist) and has been shown to have low binding affnity to CB1 receptor^27, 28^, thus the use of CB1-KO mice will exclude its possible binding to CB1 receptor. The complete procedures for T1117 binding are provided in *Supplmentary Information*.

### Experiment 4: In vivomicrodialysis in P-rats

This experiment was designed to determine whether and how systemic administraton of O-1602, a GPR55 agonist, alters extracellular DA or glutamate levels in the nucleus accumbens (NAc). We used alcohol-preferring (P-rats) in this experiment because P-rats were used to evaluate the effects of O-1602 on nicotine self-administration due to their high vulnearability to nicotine as we reported previously^29^.The complete microdialysis procedures are described in *Supplementary Information*.

### Experiment 5: D^9^-THC-induced triad effects

This experiment was designed to determine whether activation of GPR55 by O-1602 alters 30 mg/kg of D^9^-THC-induced analgesia, hypothermia and catalepsy. The complete triad experimental procedures are described in *Supplementary Information*.

### Experiment 6: Intravenous drug self-administration in rats

This experiment was designed to determine whether O-1602 is able to block intravenous cocaine or nicotine self-administration. The complete drug self-administration procedures are described in *Supplementary Information*.

### Experiment 7: Intravenous nicotine self-administration in WT and GPR55-KO mice

This experiment was designed to determine whether the effects produced by O-1602 are mediated by activation of GPR55. The complete drug self-administration procedures are described in *Supplementary Information*.

### Experiment 8: Oral sucrose self-administration

This experiment was designed to determine whether O-1602 also alters operant food-taking behavior or nature reward. The complete sucrose self-administration procedures are described in *Supplementary Information*.

### Experiment 9: open-field Locomotion

In this experiment, we examined whether O-1602 alters open-field locomotion in order to determine whether it produces sedation or locomotor impairment. The complete locomotor test procedures are described in *Supplementary Information*.

### Experiment 10: Optical Intracranial Self-Stimulation (oICSS)

This experiment was designed to determine, first, whether O-1602 alters DA-dependent oICSS, and second, whether it produces rewarding or aversive effects by itself in order to determine its abuse potential or unwanted side-effects. The complete oICSS procedures are described in *Supplementary Information*.

### Data analysis

Data analyses and graphing were accomplished using SigmaPlot software (version 13.0, Systat Software Inc., CA, USA). One-way repeated measures (RM) ANOVAs were used for comparing the effects of treatment on self-administration. Two-way RM ANOVAs were performed for analyzing the effect of treatments on locomotion, extracellular DA/glutamate, and oICSS behavior. Graphs were made based on the results reported as mean ± SEM. p < 0.05 was defined as a statistically significant difference.

## Results

### GPR55 mRNA expression in glutamate, not dopamine, neurons

**Figure 1:**
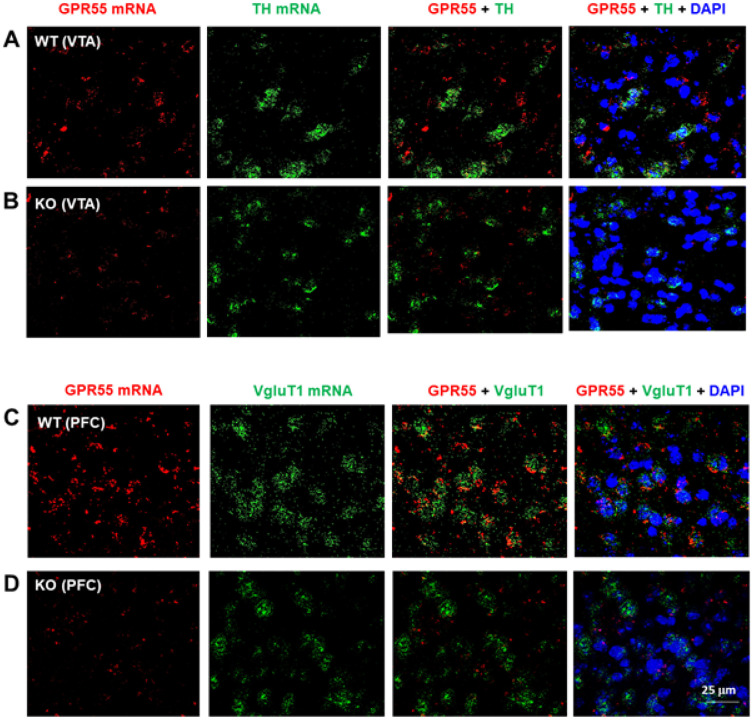
GPR55 RNAscope ISH in WT and GPR55-KO mice. **A/B**: GPR55 mRNA was detected in the VTA in WT mice **(A)**, but not in GPR55-KO mice (**B**). However, GPR55 did not show colocalization with TH mRNA, a DA neuronal marker, in the VTA of WT mice (**A**). **C/D**: High density GPR55 mRNA was also detected in the PFC of WT mice (**C**), but not in GPR55-KO mice (**D**). Notably, GPR55 mRNA colocalizes with VgluT1 mRNA, a cortical glutamatergic neuronal marker, in the PFC of WT mice (**A**). (Also see Figs. S1-S2)

To determine the cellular distributions of GPR55 in the brain, we rst used RNAscope ISH assays to examine GPR55 transcript (mRNA) expression in brain DA neurons and glutamate neurons. Figure 1 shows GPR55 mRNA staining, illustrating that GPR55 is not colocalized with tyrosine hydroxylase (TH) in DA neurons in the ventral tegmental area (VTA) of the midbrain (Fig. 1A), but it is colocalized with VgluT1 mRNA in the prefrontal cortex (PFC) glutamate neurons (Fig. 1C). Genetic deletion of GPR55 almost completely blocked GPR55 mRNA signal in the VTA (Fig. 1B) and the PFC (Fig. 1D). We also examined co-localization of GPR55 and DA transporter (DAT, another DA neuronal marker) mRNAs in the VTA, but failed to detect their colocalization (Fig. S1). Figure S1-A shows the GPR55 gene structure and the deleted region in GPR55-knockout mice. Figure S2 shows a high magnification image from Fig. 1C, illustrating clear GPR55 and VgluT1 colocalization in cortical glutamate neurons. Quantitative cell counting data indicated that ~ 90% (91.65 ± 12.43%) of cortical glutamate neurons express GPR55, while in the VTA, only ~ 10% (10.42 ± 2.14%) of DA neurons show GPR55 signal.

We also examined GPR55 mRNA expression in other brain regions. We detected similar GPR55 and VgluT1 colocalization in the hippocampus and thalamus, but not in the striatum (Fig. S3).

### GPR55-immunostaining is not GPR55-specific

Next, we used IHC assays to detect GPR55 protein expression in the regions where mRNA was detected. We used two commercially available GPR55 antibodies: a polyclonal anti-GPR55 antibody (Abcam), which targets the C-terminus of human GPR55, and another polyclonal anti-GPR55 antibody with an undisclosed epitope (Cayman Chemicals). Figures S4 and S5 shows the representative GPR55-immunostaining images, illustrating that GPR55-like signal was detected in the VTA, but it was not colocalized with TH-immunostaining in DA neurons when using either the Abcam GPR55 antibody (Fig. S3) or the Cayman GPR55 antibody (Fig. S4). However, the GPR55-immunostaining is not highly specific as it is still detectable in either GPR55-KO mice (Fig. S4-B) or in the presence of either of the GPR55 antibody immune peptides (Fig. S4-C; Fig. S5-B). These findings suggest that both the antibodies against the human or bovine GPR55 are not suitable to detect GPR55 receptor proteins in mice although human GPR55 show 75% and 78% homology with the rat and mouse GPR55 proteins, respectively^9^.

### Flfluorescent cannabinoid ligand binding reveals GPR55 expression in glutamate, not DA, neurons

**Figure 2:**
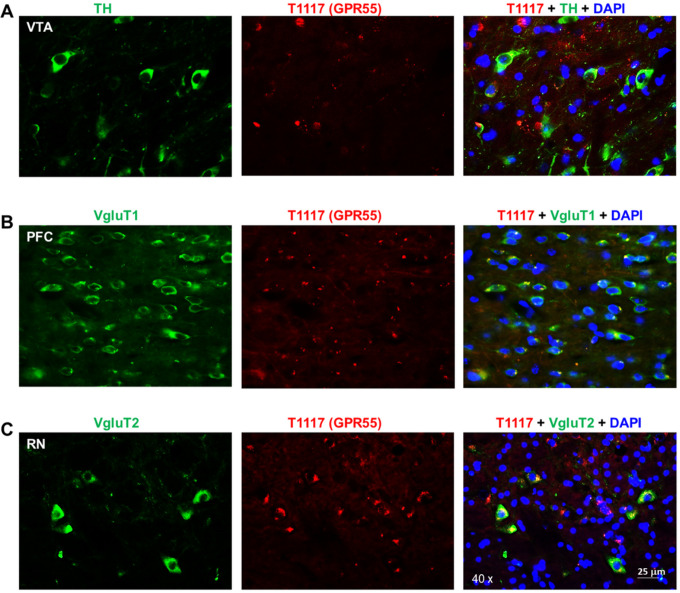
T1117 fluorescent ligand binding results in CB1-KO mice, indicating that T1117 binding signal is not colocalized with TH-immunostaining in VTA DA neurons (**A**), but it is colocalized with VgluT1- or VgluT2-immunostaining in glutamate neurons in the PFC) (**B**) and red nucleus (RN) (**C**).

**Figure 3:**
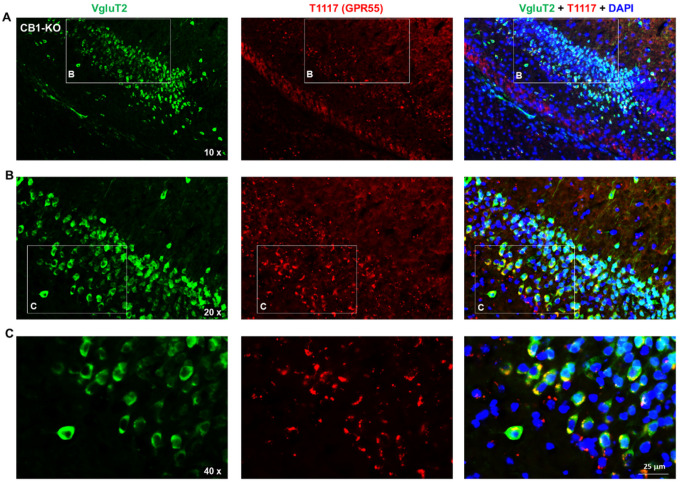
fluorescent ligand (T1117) binding in the hippocampus taken under different magni cations, indicating that T1117-labeled GPR55 is co-localized with VgluT2-immunostaining in hippocampal glutamate neurons of CB1-KO mice

To further validate our RNAscope findings on GPR55 expression in glutamate versus DA neurons, we then used a fluorescent cannabinoid ligand – Tocrifluor T1117 (T1117), an analog of AM251 (a selective CB1 receptor antagonist) – to detect GPR55 expression. Because T1117 is not a selective GPR55 agonist and also has low binding affinity to the CB1 receptor^27, 28^, we used CB1-KO mice to exclude its binding to the CB1 receptor in this assay. There are two types of glutamate neurons that express VgluT1 mainly in the cortex and VgluT2 mainly in the subcortical brain regions such as the hippocampus and thalamus^30^.Therefore, we used two different glutamatergic neuronal markers (e.g., VgluT1 and VgluT2 antibodies) to identify glutamate neurons in this study. Figure 2 shows representative T1117 binding in the VTA, PFC, and midbrain red nucleus (RN), illustrating that T1117 did not show co-localization with TH in VTA DA neurons (Fig. 2A), but showed clear T1117-VgluT1 colocalization in PFC glutamate neurons (Fig. 2B) and T1117-VgluT2 colocalization in red nucleus glutamate neurons (Fig. 2C). Notably, T1117 fluorescent signal is also detected in other non-DA neurons in the VTA (Fig. 2A) or non-glutamate neurons in the red nucleus (Fig. 2-C). Figure 3 shows the representative T1117 binding images in the hippocampus under different magnifications (20×, 40×, and 60×), indicating that T1117 and VgluT2 colocalization in the majority of VgluT2-positive glutamate neurons in the hippocampus of CB1-KO mice. We did not use GPR55-KO mice to determine the signal specificityas T1117 also binds to CB1 receptor in GPR55-KO mice and double CB1-KO and GPR55-KO mice are currently not available. Together, these ligand binding data, combined with our data from RNAscope ISH and IHC assays, support a conclusion that GPR55 is mainly expressed in cortical and subcortical glutamate neurons, but it is not expressed in midbrain DA neurons.

### O-1602 elevates extracellular glutamate, not DA, in the NAc

Next we used *in vivo* brain microdialysis (Fig. 3A) to examine whether activation of GPR55 alters glutamate or DA release in the NAc, a critical brain region involved in cannabis action and drug abuse^2, 5^.We found that O-1602, at 3 and 10 mg/kg, failed to alter extracellular DA (Fig. 3B), but produced a transient increase in extracellular glutamate levels in the NAc (Fig. 3C). An one-way RM ANOVA for the data shown in gray boxes did not reveal a O-1602 treatment main effect on extracellular DA after 3 mg/kg (Fig. 3B, F_4,20_=1.16, p > 0.05) or 10 mg/kg (Fig. 3B, F_4,20_=0.51, p > 0.05) O-1602 administration. However, the one-way RM ANOVA revealed a significant O-1602 treatment main effect in extracellular glutamate after 10 mg/kg (Fig. 3C, F_2,24_=4.17, p = 0.01), but not after 3 mg/kg (Fig. 3C, F_4,24_=1.76, p > 0.05), O-1602 administration.

### O-1602 failed to alter D^9^-THC-induced triad effects

We have recently reported that genetic deletion of GPR55 enhanced D^9^-THC-induced analgesia, hypothermia, and catalepsy^22^, suggesting that GPR55 activation may inhibit cannabinoid action. Therefore, we proposed that GPR55 agonists may have therapeutic effects against cannabinoid action. To test this hypothesis, we first observed the effects of O-1602 (a potent GPR55 agonist) on a high dose (30 mg/kg) of D^9^-THC-induced classical triad effects. We found that pretreatment with O-1602 (10, 20 mg/kg, i.p., 15 min prior to D^9^-THC) failed to alter 30 mg/kg D^9^-THC-induced analgesia, hypothermia, or catalepsy (Fig. S6) although a trend toward an increase in analgesia and hypothermia compred to the vehicle group. However, two-way RM ANOVA did not reveal a significant O-1602 treatment main effect in D^9^-THC-induced analgesia (Fig. S6-A, F_7,2_ =0.994, p > 0.05), D^9^-THC-induced hypothermia (Fig. S6-B, F_7,2_=0.441, p > 0.05), or D^9^-THC-induced catalepsy (Fig. S6-C, F_7,2_=2.31, p > 0.05).

### O-1602 inhibits nicotine, not cocaine, self-administration in rats

We have recently reported that elevation of extracellular glutamate in the NAc by blockade of glial GLT-1 inhibits cocaine self-administration^31^. Next, we examined whether O-1602, which transiently elevates extracellular glutamate in the NAc (Fig. 3), also inhibits intravenous cocaine self-administration in rats or mice. Figure 5 (A, B) shows that systemic administration of O-1602, at 10 mg/kg and 20 mg/kg, failed to alter cocaine self-administration under FR2 reinforcement in rats (Fig. 5A, one-way RM ANOVA, F_2,14_=3.69, p > 0.05) or under FR1 reinforcement in wildtype mice (Fig. 5B, one-way ANOVA, F_2,20_=1.40, p > 0.05), suggesting that O-1602 has no significant effect on cocaine self-administration.

We then examined the effects of O-1602 on intravenous nicotine self-administration as it was reported that systemic or intracerebroventricular administration of GPR55 agonists inhibied conditioned place preference to nicotine^25, 26^. Here we chose selectively bred alcohol-preferring rats (P-rats), as P-rats displayed significantly higher vulnerability than Long-Evens (LE) rats in nicotine self-administration in our previous report^29^. We found that O-1602 (3, 10, or 20 mg/kg, 15 min prior to nicotine SA session) produced a dose-dependent reduction in either the total number of nicotine infusions or the rate of nicotine self-administration (i.e., nicotine infusions/hr) (Fig. 5-C, D). This inhibitory effect was blocked by co-administration of CID 16020046 (CID), a selective GPR55 antagonist. CID alone, at 5 or 10 mg/kg, failed to alter nicotine self-administration (Fig. 5-E, F). A one-way RM ANOVA revealed a significant main effect of O-1602 treatment in the number of infusions (Fig. 5C, F_4,23_ =3.28, p < 0.05) and the rate of infusions (Fig. 5D, F_4,23_ =4.75, p < 0.01). Post-hoc individual group comparisons indicated a significant reduction in nicotine self-administration after 20 mg/kg O-1602, compared to the vehicle control group.

To determine whether O-1602 also alters motivation for nicotine taking and seeking, we observed the effects of O-1602 on nicotine self-administration under a PR reinforcement schedule. Figure 5 (E, F) shows that pretreatment with O-1602 dose-dependently inhibited PR nicotine self-administration as assessed by the number of nicotine infusions (Fig. 5E, F_5,42_ =12.90, p < 0.001) or PR break-point (Fig. 5F, F_5,42_=5.41, p < 0.001).

### O-1602 inhibits nicotine self-administration in WT, not GPR55-KO, mice

To further determine whether the above action produced O-1602 is mediated by activation of GPR55, we used GPR55-KO mice in the self-administration experiment. Figure 6 (A, B) shows that GPR55-KO mice did not differ in nicotine self-administration compared to WT mice (e.g., between the vehicle control groups). However, systemic administration of O-1602 (10, 20 mg/kg, i.p.) produced a robust reduction in nicotine self-administration in WT mice as assessed by either the total number of nicotine infusions (Fig. 6A, F_3,21_=11.32, p < 0.001) or the rate of nicotine self-administration (Fig. 6B, F_3,21_=16.25, p < 0.001), but not in GPR55-KO mice (Fig. 6A, F_3,18_=0.71, p > 0.05; Fig. 6B, F_3,18_=1.06, p > 0.05). Figure 6 (C, D) shows representative nicotine self-administration (infusion) records after vehicle or O-1602 treatment in WT mice, indicating that 10 mg/kg of O-1602 significantlyinhibited nicotine self-administration and altered the patterns of self-administration from a regular, evenly-distributed pattern to an irregular, extinction-like pattern, suggesting that GPR55 agonism inhibits nicotine reward.

### O-1602 has no effect on oral sucrose self-administration or open-field locomotion

To determine whether the O-1602-induced reduction in nicotine self-administration was due to treatment-induced locomotor impairment, we observed the effects of O-1602 on open-field locomotion and non-drug (sucrose) self-administration. We found that systemic administration of the same doses of O-1602 neither altered sucrose self-administration (Fig. S7-A, F_2,13_=1.05, p > 0.05; Fig. S7-B, F_2,13_=1.38, p > 0.05) nor altered open-field locomotion (Fig. S7-C, main effect of O-1602 treatment, F_3,21_=0.25, p > 0.05; treatment × time interaction, F51,357=0.38 p > 0.05), suggesting that O-1602 selectively inhibits nicotine self-administration.

### O-1602 is not rewarding or aversive by itself

**Figure 4:**
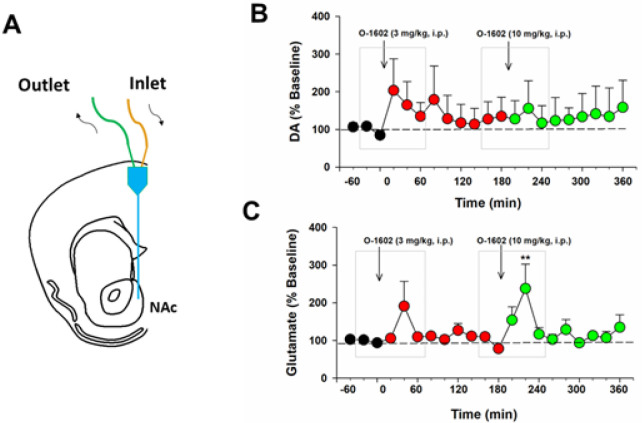
Effects of O-1602 on extracellular DA and glutamate levels in the NAc of P-rats. **A**: A diagram showing a microdialysis probe targeting the NAc-shell. **B/C**: Systemic administration of O-1602 failed to alter extracellular DA in the NAc (**B**), while it dose-dependently elevated extracellular glutamate levels in the NAc (C) in rats after O-1602 administration. **p<0.01, compared to the baseline before O-1602 administration.

**Figure 5:**
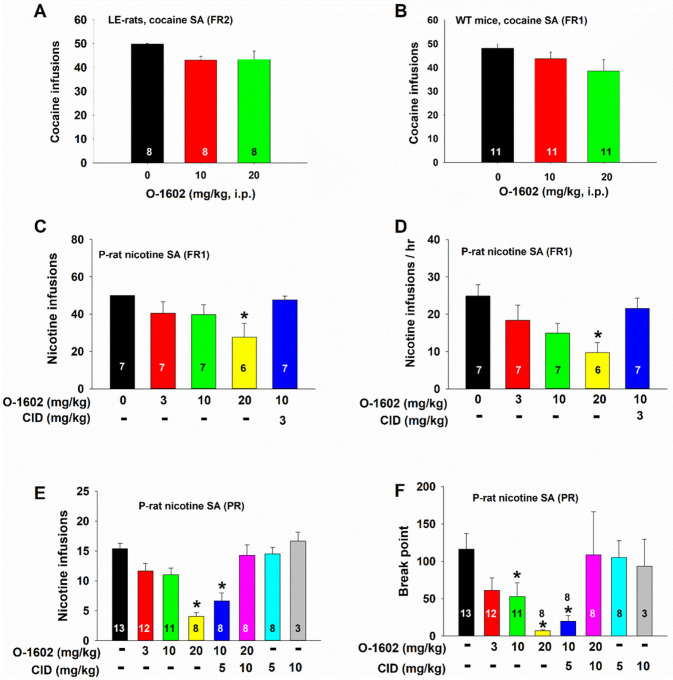
Effects of O-1602 on intravenous cocaine or nicotine self-administration (SA). **A/B**: Systemic administration of O-1602 failed to alter cocaine self-administration under FR1 or FR2 reinstatement schedules in Long-Evan rats **(A)** or wildtype mice (**B**). **C/D**: O-1602 dose-dependently inhibited nicotine SA under FR1 reinforcement as assessed by the total number of nicotine infusions (**C**) or the rate of nicotine infusions (**D**). E/F: O-1602 also inhibited nicotine SA under progressive-ratio (PR) reinforcement as assessed by the number of infusions (**E**) or break-point for nicotine SA (**F**). Pretreatment with CID 16020046 (CID), a selective GPR55 antagonist, blocked action produced by O-1602. *p<0.05, compared to the vehicle control group

**Figure 6:**
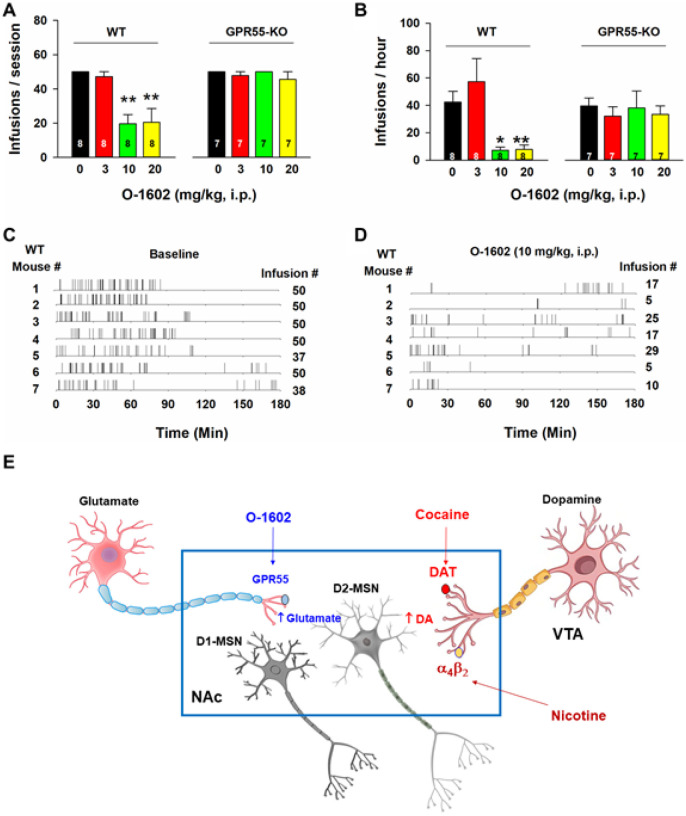
Effects of O-1602 on nicotine self-administration in WT and GPR55-KO mice. **A/B**: Systemic administration of O-1602 (10 and 20 mg/kg) significantly inhibited nicotine self-administration in WT mice, but not in GPR55-KO mice. **C/D**: Representative records of nicotine infusions after the vehicle (**C**) or O-1602 administration (**D**), illustrate that O-1602, at 10 mg/kg, caused cessation of self-administration. E: A proposed working hypothesis explaining how GPR55 agonism reduces nicotine self-administration. Briefly, cocaine or nicotine may increase extracellular DA levels in the NAc by blockade of DA transporter (DAT) or activation of a_4_b_2_ nicotinic receptors located on midbrain DA neurons or NAc DA terminals, respectively, which has been thought to underlie intravenous drug self-administration. Activation of GPR55 expressed on glutamate terminals projected from the cortex and hippocampus increases glutamate release in the NAc, which subsequently counteracts DA effects on postsynaptic medium-spiny neurons (MSNs), particularly in D2 receptor-expressing MSNs (D2-MSNs) where glutamate and DA produce opposite effects on neuronal activity. ** p<0.01, compared to the vehicle control group.

Lastly, we examined whether O-1602 produces rewarding or aversive effects by itself as assessed by optical intracranial self-administration (oICSS). We have previously reported that cocaine or oxycodone produces reward-enhancing^32, 33^, while cannabinoids such as D^9^-THC and WIN55-212-2 produces reward-attenuation (aversive) effects in oICSS in DAT-Cre mice^33^. Using the same approaches (Fig. S8-A ~ D), we found that optogenetic stimulation of VTA DA neurons produced robust oICSS behavior in a stimulation frequency-dependent manner (Fig. S8-E, F). O-1602, at the same doses as used in nicotine self-administration, did not significantly alter oICSS (Fig. 6F), suggesting that GPR55 itself is not rewarding or aversive. It also suggests that O-1602 does not alter DA-dependent behavior. This is finding is consistent with our findings that GPR55 is not identified in midbrain DA neurons (Figs. 1, 2; Figs. S4 and S5) and O-1602 also failed to alter DA release in the NAc (Fig. 4). Figure 6E shows a proposed hypothesis through which O-1602 elevates extracellular glutamate that subsequently counteracts the action produced by nicotine-enhanced DA mainly in D2-expressing medium-spiny neurons (D2-MSNs).

## Discussion

In this study, we systematically investigated the cellular distributions and function of GPR55 in the mouse brain. We found that: 1) GPR55 mRNA is highly expressed in VgluT1^+^ glutamate neurons in the PFC, hippocampus and thalamus, but not in midbrain DA neurons. 2) Two polyclonal GPR55 antibodies detected GPR55-like immunostaining, but the signal is not GPR55-speci c. 3) Using a fluorescent CB1-GPR55 ligand (T-1117), we detected GPR55 binding in both VgluT1^+^ glutamate neurons in the PFC and VgluT2^+^ glutamate neurons in hippocampus and red nucleus, but not in VTA DA neurons, in CB1-KO mice.4) Systemic administration of O-1602, a GPR55 agonist, dose-dependently increased glutamate, but not DA, release in the NAc, as assessed by in vivo microdialysis. 5) O-1602 dose-dependently inhibited nicotine (not cocaine) self-administration. In contrast, O-1602 neither altered D^9^-THC-induced triad effects nor open-field locomotion or sucrose self-administration. In oICSS, it is not rewarding or aversive by itself. Taken together, these findings suggest that GPR55 may constitute a new therapeutic target for the treatment of nicotine use disorders.

### Identification of GPR55 expression in glutamatergic neurons

The first important finding in this study is the identification of GPR55 mRNA in cortical and subcortical glutamate neurons, but not in midbrain DA neurons. This finding is consistent with a previous report indicating that GPR55 mRNA is colocalized with the neuronal marker NeuN, but not with an astrocytic marker GFAP or microglial marker Iba1 in the striatum^15^. Notably, a high density of GPR55 mRNA was detected in the cortex, while much lower GPR55 mRNA was detected in subcortical regions such as the hippocampus, thalamus, VTA, red nucleus, and striatum.

Unexpectedly, we found that the GPR55-immunostaining detected by two anti-GPR55 antibodies is not highly GPR55-speci c, as it is still detectable in GPR55-KO tissues or in WT mice in the presence of specific immune peptides. This contrasts with two previous reports indicating specific GPR55-immunostaining in the hippocampus and striatum in WT but not in GPR55-KO mice, using an anti-GPR55 antibody provided by Ken Mackie^17, 18^. The reasons for these conflicting findings are unclear. It may be related to the different epitopes of the antibodies. For example, the Abcam antibody we used in this study targets the C-terminal of human or bovine GPR55, while the epitopes of the Ken Mackie antibody^18^ and the Cayman antibody we used in this study are undisclosed. In addition, it was recently reported that GPR55 is colocalized with substance P in striatal MSNs using another anti-GPR55 antibody (provided by Bioss). This antibody targets the transmembrane domains of hGPR55^16^. However, the GPR55 specificity of this antibody is not tested in GPR55-KO mice.

Due to the concern of the antibody specificity, we then used a fluorescent CB1-GPR55 ligand (T1117) to examine GPR55 binding in CB1-KO mice. We had the same findings as those from RNAscope ISH. High-density GPR55 binding was detected in cortical VgluT1^+^ glutamate neurons and subcortical VgluT2^+^ glutamate neurons in the hippo campus, thalamus and red nucleus, but not in midbrain DA neurons. This finding is consistent with a previous report that the GPR55 is colocalized with VgluT1 in the hippocampus by double-label IHC in which the Ken Mackie anti-GPR55 antibody was used^17^. Given that all three approaches (RNAscope ISH, IHC, and fluorescent T1117 binding) did not detect GPR55 signals in VTA DA neurons, we concluded that GPR55 may not directly regulate DA neuron activity and DA-related behavior. In contrast, all the above findings support GPR55 expression in glutamate neurons, suggesting that glutamatergic GPR55 may play an important role in GPR55 function under physiological conditions and pharmacological action produced by GPR55 ligands in the brain.

### GPR55 agonist failed to alter high dose Δ^9^-THC-induced triad effects

As stated above, GPR55 has been thought to be a putative “CB3” receptor. However, little is known about the functional role of GPR55 in cannabinoid action. In this study, we found that pretreatment with O-1602 failed to alter Δ^9^-THC-induced classical triad effects (analgesia, hypothermia and catalepsy). This finding is in contrast with our previous finding that GPR55-KO mice displayed enhanced tetrad in responses to Δ^9^-THC or WIN55,212–2^22^. The mechanisms underlying these inconsistent findings are unknown. It may be related to the very high Δ^9^-THC dose (30 mg/kg). Thus, higher O-1602 doses may be required to alter Δ^9^-THC action in the triad assays. Another possibility is that Δ^9^-THC, at high doses, may bind to other cannabinoid receptors such as CB1, CB2 and PPARs in distinct brain regions^5, 34^, producing potent analgesia, hypothermia and catalepsy that compromise action produced by the GPR55 agonist O-1602

### GPR55 agonism failed to alter cocaine, but inhibited nicotine self-administration

Another important finding in this study is that GPR55 agonism significantly inhibited nicotine, but not cocaine, self-administration, an effect that was blocked by GPR55 antagonism or genetic deletion of GPR55, suggesting that GPR55 may constitute a new therapeutic target for the treatment of nicotine use disorder. The reduction in nicotine self-administration is unlikely due to sedation or locomotor impairment because O-1602, at the same doses, failed to alter oral sucrose self-administration or open-field locomotion. These findings are consistent with previous reports that O-1602, at much lower doses (0.4 ~ 5 mg/kg, i.p.), not only attenuated CPP response to morphine or nicotine but also attenuated naloxone-precipitated opioid withdrawal syndromes^24, 25^.

We note that O-1602 is not a highly selective GPR55 agonist^9^. It is also a biased GPR18 agonist^35, 36^, suggesting that non-GPR55 mechanisms may also contribute to the pharmacological action produced by O-1602 in this study. This possibility is unlikely as genetic deletion of GPR55 in GPR55-KO mice almost completely blocked the effects of O-1602 on nicotine self-administration, supporting critical involvement of GPR55 in the pharmacological action of O-1602.

### Glutamate mechanisms may underlie GPR55 modulation of nicotine self-administration

It is unknown exactly how GPR55 modulates nicotine self-administration. Given that GPR55 agonism may increase glutamate, but not DA, release in the NAc, we hypothesized that an enhanced glutamate transmission in the NAc may underlie the antagonism of O-1602 on drug self-administration (Fig. 6E).This is supported by several lines of evidence. First, activation of GPR55 increases intracellular Ca^++^ levels in neurons in the hippocampus, substantia nigra, and dorsal root ganglia^16, 17, 37^, and facilitates glutamate release in the NAc (present study) and hippocampus^17^. Second, optical stimulation of glutamate terminals in the NAc projected from the paraventricular nucleus of the thalamus is not rewarding, but reward-attenuating or aversive^38^. And third, the elevation of extracellular glutamate levels in the NAc by blockade of glial glutamate transporters inhibits cocaine self-administration and cocaine intake via an action on postsynaptic medium-spiny neurons^31^.

It is well documented that drug self-administration is maintained mainly by a DA-dependent mechanism^39, 40^. Cocaine increases extracellular DA level in the NAc by blockade of DA transporter (DAT), while nicotine increases NAc DA release mainly by activation of a_4_b_2_ nicotinic receptors located on midbrain DA neurons^39, 41^. DA subsequently activates D1 receptor-expressing medium-spiny neurons (D1-MSNs) via excitatory Gs-coupled proteins, while it inhibits D2-MSNs via inhibitory Gi-coupled proteins, leading to rewarding effects^42^. Because O-1602 increases NAc glutamate release, which may functionally counteract DA action in D2-MSNs^31^, we therefore proposed that this glutamate mechanism may in part explain how GPR55 agonism produces inhibitory effects on cocaine or nicotine self-administration observed in the present study (Fig. 6E). We note that O-1602 failed to alter cocaine self-administration in this study. The precise reasons are unknown. There are two possibilities. First, cocaine-enhanced extracellular DA in the NAc appears to be more potent and dynamic (fast-onset and short-acting) than nicotine-enhanced DA (slow-onset, long-acting)^29, 43^, thus, O-1602-induced increase in extracellular glutamate may be able to counteract action produced by nicotine-enhanced DA, but not by cocaine-enhanced DA during self-administration. Second, increased extracellular glutamate derived from presynaptic terminals (by O-1602 in the present study) versus from extrasynaptic space by blockade of glial glutamate transporters^31^ may differentially modulate nicotine versus cocaine self-administration. Clearly, more studies are required to further address this issue.

We note another report that intra-ventral hippocampus (intra-vHipp) microinjection of PEA, an endogenous GPR55 and PPARa agonist^44, 45^ increased ring and bursting activity of VTA DA neurons, an effect that was blocked by a selective GPR55 antagonist CID16020046^46^, suggesting that a DA-dependent mechanism may underlie GPR55 action. However, in the present study, we did not detect GPR55 expression in VTA DA neurons. In this study, we neither detected GPR55 expression in VTA DA neurons nor detected O-1602 modulation of extracellular DA and DA-dependent oICSS behavior, suggesting that an indirect mechanism may underlie the effects of intra-vHipp PEA-enhanced DA neuron ring. This is supported by the findings in the same report that an NMDA receptor antagonist can block intra-vHipp PEA-induced increase in VTA DA neuron ring and the local PEA-enhanced DA failed to alter morphine-induced CPP^46^.

In summary, in this study, we systematically examined the cellular distributions of GPR55 in the mouse brain and its functional role in cannabinoid action and substance use disorders. We found that GPR55 is mainly expressed in cortical and subcortical glutamate neurons but not in midbrain DA neurons. Activation of GPR55 by O-1602 selectively increased glutamate release in the NAc and dose-dependently inhibited nicotine self-administration in rats and mice, but did not alter cocaine or Δ^9^-THC action, suggesting that a glutamate mechanism may underlie the pharmacological action of GPR55 agonism in nicotine taking and seeking behavior. Thus, GPR55 deserves further research as a new therapeutic target for treating nicotine use disorders.

## Data Availability

The raw data in this manuscript is available upon request
